# Subcortical Facilitation of Behavioral Responses to Threat

**DOI:** 10.1038/s41598-017-13203-8

**Published:** 2017-10-12

**Authors:** Mark D. Vida, Marlene Behrmann

**Affiliations:** 1 Rotman Research Institute, Baycrest Health Sciences, Toronto, Canada; 2 Department of Psychology and Center for the Neural Basis of Cognition, Carnegie Mellon University, Pittsburgh, USA

## Abstract

Behavioral responses to threat are critical to survival. Several cortical and subcortical brain regions respond selectively to threat. However, the relation of these neural responses and their underlying representations to behavior is unclear. We examined the contribution of lower-order subcortical representations to behavioral responses to threat in adult humans. In Experiments 1 and 2, participants viewed pairs of images presented to the same eye or to different eyes. We observed a monocular advantage, which indicates subcortical facilitation, for ancestral threats (snakes, spiders), but not for modern threats, positive images, or neutral images. In Experiment 3, we presented pairs of snakes or neutral images into the temporal or nasal hemifield. For snakes only, we observed a temporal hemifield advantage, which indicates facilitation by the retino-tectal subcortical pathway. These results advance the current understanding of processing of threat by adult humans by revealing the characteristics of behaviors driven by a lower-order neural mechanism that is specialized for the processing of ancestral threats. The results also contribute to ongoing debates concerning the biological generality of neural mechanisms for processing of complex, emotionally-relevant stimuli by providing evidence for conservation of lower-order neural mechanisms for processing of ancestral threats across both ontogeny and phylogeny.

## Introduction

Adult humans detect and respond more rapidly to threatening images than to non-threatening images, whether the images depict ancestral threats^1–7^ (e.g., snakes, spiders) or modern threats^8,9^ (e.g., guns). This enhanced behavioral performance for threatening stimuli may be important for avoiding danger in real-world situations, and may reflect specialization of neural mechanisms for this purpose, resulting from selection of individuals proficient in detecting and/or responding to visual threat^10–12^. Several authors have proposed that behavioral responses to threat may reflect an evolved subcortical neural mechanism that is conserved across phylogeny and ontogeny^11–15^. However, many cortical and subcortical brain regions appear to be selectively involved in representing threatening stimuli. It remains unclear what information about these stimuli is represented in each region, and how these neural representations contribute to behavioral responses to threat^13,15^. Indeed, much of the literature in this domain is cortico-centric, and perhaps because of the difficulty in measuring from these small, deep structures, few studies have addressed the precise role of subcortical structures in driving behavioral responses to threat in humans. In the current study, we used monocular/dichoptic behavioral paradigms to investigate the contribution of the subcortical visual pathway to behavioral responses to threat in adult humans.

Neuroimaging and neuropsychology studies in adult humans have provided evidence that both subcortical and cortical regions are involved to at least some degree in processing of visual threat. Viewing of threatening images appears to selectively activate several subcortical regions, including the amygdala, superior colliculus, and pulvinar^16–19^. Also, one study has demonstrated that the extent of attentional bias toward fearful faces (which can signal threat under at least some circumstances) is positively correlated with activity in the pulvinar, and with the degree of functional connectivity of pulvinar with fronto-parietal cortex^20^. Furthermore, neuropsychology studies have demonstrated that damage to the pulvinar disrupts interference from threatening images on goal-directed behavior^21^, and that bilateral damage to amygdala produces a selective impairment in the recognition of fearful facial expressions^22^. However, the results of these lesion studies should be interpreted with caution, as changes in behavior associated with damage to a specific region may not necessarily reflect the function of that region^23^. With respect to cortical regions, neuroimaging studies in adult humans indicate that threatening images selectively activate prefrontal^20,24,25^, anterior cingulate^24^, and orbitofrontal^26^ cortices. Together, these results suggest that both subcortical and cortical networks may be involved in processing of visual threat. However, given that the subcortical and cortical pathways are highly interconnected^13^, and that most of these studies did not measure the relationship between neural activity in the intact brain and behavioral responses to threat, it remains unclear what information about threatening stimuli is represented in the subcortical visual pathway, and how subcortical representations contribute to behavioral responses to threat in human adults.

As with humans, non-human primates respond more rapidly to at least some types of threatening images than to neutral images^27,28^. Also, viewing threatening images appears to activate many of the same subcortical and cortical brain regions in human and non-human primates^29–31^. One prominent account of these similarities suggests that both human and non-human primates share a neural mechanism for rapid detection and avoidance of ancestral threats (e.g., snakes), which may be based in the subcortical visual pathway^11,12,14,15^. However, comparisons of behavioral responses to specific types of threats between human and non-human primates suggest at least some degree of divergence. Humans are consistently faster at detecting snakes than neutral targets^3–7^, and are faster at detecting spiders than neutral targets under at least some circumstances^2,32^. In contrast, non-human primates are faster at detecting snakes than neutral animals^28^, but are not faster at detecting spiders than neutral animals^27^. Also, whereas both human and non-human primates commonly fear snakes, fear of spiders is common only in humans^27^. With respect to the question of conservation over phylogeny, it is of interest whether there is subcortical facilitation of behavioral responses to threat in adult humans, which would suggest that the lower-order neural mechanism proposed to facilitate threat processing in both human and non-human primates is active in humans^15^. Of interest too, is whether there is subcortical facilitation for both snakes and spiders in adult humans, a result that would provide further evidence regarding the degree of divergence between human and non-human primates.

Perceptual biases toward threatening stimuli have also been observed in studies of infant and child development. Infants as young as 5 months of age look longer at threatening stimuli than at non-threatening stimuli^32^, and infants as young as 8 months and young children detect threatening images (e.g., snakes, angry faces) faster than non-threatening images (e.g., flowers and happy faces)^2,33,34^. Given that infants as young as 5 months of age are likely to have had little experience with threats such as snakes, these results may suggest that perceptual biases toward threatening stimuli are not entirely learned, and could reflect a subcortical mechanism present from birth^13^. That these perceptual biases remain in adulthood could suggest that this subcortical mechanism continues to function beyond infancy. However, it is also possible that such a mechanism would be supplemented or replaced by cortical mechanisms after childhood, and would therefore not be entirely conserved over ontogeny^13^. Hence, with respect to the question of conservation over ontogeny, it is of interest whether the subcortical visual pathway contributes to facilitation of behavioral responses to threat in adult humans, which would suggest that the subcortical mechanism proposed to guide threat processing early in development continues to function in adulthood.

In the current study, we investigated three important and unanswered questions concerning processing of threat in adult humans: 1) Does the subcortical visual pathway facilitate behavioral responses to threat? 2) Which types of threats receive subcortical facilitation? 3) Which parts of the subcortical visual pathway are involved in facilitation of behavioral responses to threat? To investigate the first two questions, we used a monocular/dichoptic behavioral paradigm in which pairs of images were presented sequentially, with both images presented to the same eye or one to each eye^35^. This paradigm takes advantage of the fact that inputs from the left and right eyes are mostly segregated throughout the subcortical visual pathway, up to layer IV of primary visual cortex^36,37^, whereas inputs from the two eyes appear to be integrated to a greater extent in extrastriate cortex^38^. Two images presented to the same eye are likely to activate overlapping populations of monocular subcortical neurons, whereas two images presented to different eyes are not. Hence, a monocular advantage in responses to images within a particular category may reflect subcortical facilitation of responses for that category. This approach has been used to examine the role of the subcortical visual pathway in face recognition^35^ and processing of numerical quantity^39^. In Experiment 1, we investigated whether the subcortical visual pathway facilitates behavioral responses to ancestral threats (snakes and spiders), and to neutral images. We expected that if this facilitation were to occur, there would be a monocular advantage for snakes and spiders, but not for neutral images. Note that we classify both snakes and spiders as ancestral threats to humans, because the environment in which humans evolved included species of venomous snakes and spiders whose bites were fatal or extremely debilitating to humans^10,40–44^. In Experiment 2, we examined the specificity of the subcortical facilitation. We used the paradigm from Experiment 1 to test whether there is a monocular advantage for a modern, man-made threat (guns), and for emotionally-relevant, but non-threatening images (pleasant nature scenes).

In Experiment 3, we examined whether the retino-tectal subcortical visual pathway facilitates behavioral responses to threat. This pathway projects from the retina to the superior colliculus, pulvinar, and amygdala, in parallel with the principal geniculo-striate pathway, which projects from the retina to the lateral geniculate nucleus and primary visual cortex^13,14,45,46^. The retino-tectal pathway contains more projections from the contralateral nasal hemiretina (temporal visual hemifield) than from the temporal hemiretina (nasal visual hemifield)^47,48^. Hence, information from the temporal visual hemifield is expected to reach this pathway more readily than information from the nasal hemifield^49^. In Experiment 3, we compared behavioral responses to snakes and neutral images presented in the temporal and nasal hemifields, with the expectation that if the retino-tectal pathway selectively facilitates responses to threat, there would be a temporal hemifield advantage for snakes, with a weaker or absent temporal hemifield advantage for neutral images.

## Experiment 1

### Method

#### Participants

Participants in Experiment 1 were 35 adults (24 female) aged 18–28 years (*M* age = 20.86 years). One additional participant was tested, but was excluded from the analyses because a technical problem with the experimental setup prevented acquisition of a complete data set. Four additional participants were tested but were excluded because their accuracy or mean reaction time (see Results for description of these measures) was greater than 2.5 standard deviations away from the group mean in at least one condition. All participants had normal or corrected-to-normal visual acuity, and no history of eye problems. Protocols were approved by institutional review boards at Carnegie Mellon University. All methods were performed in accordance with the relevant guidelines and regulations. All participants provided written informed consent prior to each session, and received monetary compensation or course credit for their participation.

#### Stimuli

Stimuli in Experiment 1 were images of snakes, spiders, and neutral images from the Geneva Affective Picture Database (GAPED)^50^ (all GAPED images are available at http://www.affective-sciences.org/home/research/materials-and-online-research/research-material/). The GAPED images have been rated on several dimensions, including valence (0 to 100, with higher values being more positive) and arousal (0 to 100, with higher values indicating greater arousal)^50^. The neutral stimuli were more positive in valance (*M* valence = 55.78, *SD* valence = 6.08) and lower in arousal (*M* arousal = 24.93, *SD* arousal = 7.75) than the snakes (*M* valence = 41.52, *SD* valence = 11.17, *M* arousal = 53.63, *SD* arousal = 10.72) and spiders (*M* valence = 35.12, *SD* valence = 11.37, *M* arousal = 58.16, *SD* arousal = 10.32)^45^. Within each category, images including people, written text, or animals (neutral images only) were excluded, leaving 87 neutral images, 127 snake images and 151 spider images. To equalize the stimulus counts between categories, we randomly selected subsets of 87 snake images and 87 spider images from among the images remaining in these categories. Images were presented in grayscale against a gray background set to the mean luminance of the display. The mean luminance of each image was set to the same luminance as the background, and the RMS contrast of each image was set to 0.2. All images were presented at their original aspect ratio, at a size of 10.63 cm wide × 7.97 cm high (9.57° wide × 7.18° high at a viewing distance of 63.5 cm, see Apparatus for full dimensions).

#### Apparatus

**Figure 1:**
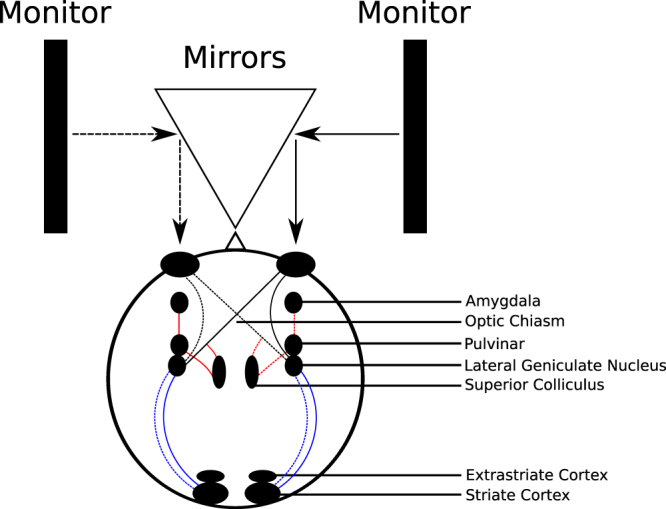
Schematic diagram of stereoscope apparatus and visual pathway with relevant cortical and subcortical regions. Each monitor provides visual input to a single eye. Dashed lines shows input from left eye and solid lines show input from right eye. From the monocularly segregated optic chiasm, the retino-tectal pathway (shown in red) projects from superior colliculus, to pulvinar and amygdala. The parallel retino-geniculate pathway (shown in blue) projects from lateral geniculate nucleus to striate and binocular extrastriate cortex. For simplicity, we depict only the input from the contralateral eye to the superior colliculus.

A chin rest was used to stabilize the participant’s head in front of a Wheatstone^51^ stereoscope. (see Fig. 1). Two ASUS VG248 (24”, 1920 × 1080 resolution, 60 Hz refresh rate) monitors were placed to the left and right of the center of the chin rest, perpendicular to the participant’s line of sight, and facing each other. Two front surface mirrors were placed in front of the participant’s left and right eyes at 45 degree angles, so that each mirror reflected the image from one of the monitors into one of the participant’s eyes. The distance from the chin rest to the center of the mirror was 8 cm, and the distance from the center of the mirror to the monitor on the same side was 55.5 cm, for a total viewing distance of 63.5 cm. Two black cardboard dividers were attached to the chin rest to block the participant’s view of the monitors, so the display would only be seen through the mirror. Participants entered responses by pressing the “F” and “H” keys on a standard computer keyboard to indicate “same” and “different” responses, respectively (see Design and Procedure for description of task).

#### Design and Procedure

At the start of each session, participants completed a calibration procedure that set the position of the fixation cross to achieve fusion of the images presented on each monitor. The fixation crosses were initially set at positions spaced widely apart, so that the participant saw two crosses. The experimenter then used a separate keyboard to gradually move the two crosses to reach positions that would produce maximum fusion of the two crosses. These positions were used for the remainder of the session.

**Figure 2:**
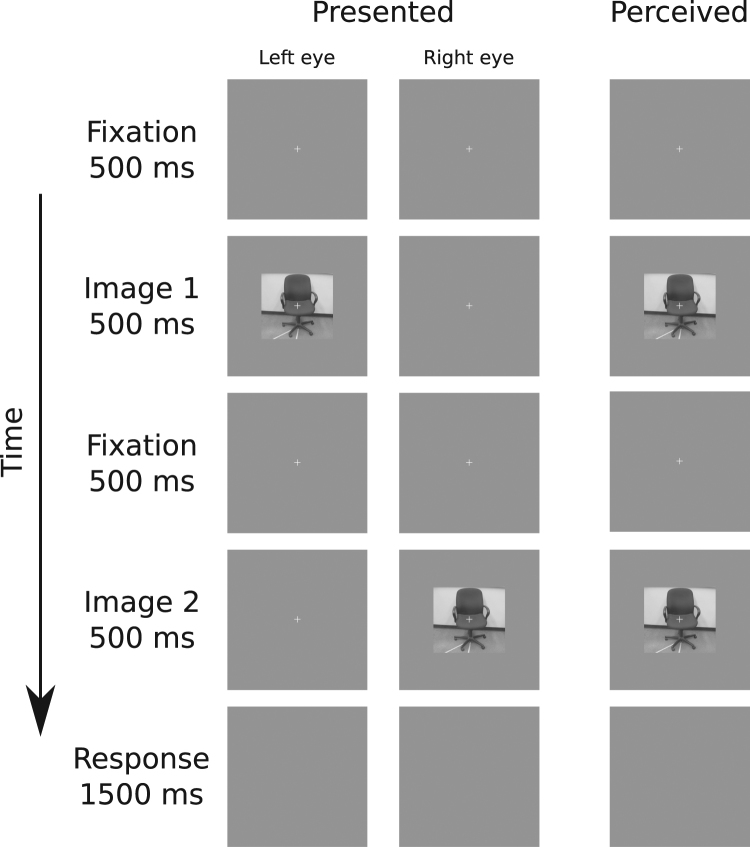
Sequence of events for an example trial in Experiment 1 or 2, in which each image was presented into a different eye. The “presented” column shows the events presented to each eye, and “perceived” column shows the participant’s fused percept. The stimulus image depicted here was not part of the stimulus set presented during the experiment, and is included here for demonstration purposes only.

After calibration, the main task began. Each participant completed two blocks of trials for each of three categories of images (neutral, snakes, spiders), for a total of six blocks. The order in which blocks were presented was randomized for each participant. Participants completed 72 trials per block. On each trial, participants viewed a pair of images randomly selected from all images available in the current category (see Fig. 2). On half of the trials, both images were presented to the same eye (half to left eye, half to right eye). The images were presented to different eyes on the other half of the trials (half starting on the left, half starting on the right). On each trial, a fixation cross appeared for 500 ms followed by the first image for 500 ms, then another fixation cross for 500 ms, then by the second image for 500 ms. Finally, a blank response screen was presented for 1.5 s. Participants were instructed to respond as quickly and accurately as possible to indicate whether the two images were the same or different. Participants were told that they could respond as soon as the second image appeared on the screen. If there was no response by 1.5 s or an incorrect response was entered, a red X was presented for 1 s. If the participant entered a correct response within the time limit, the experiment proceeded to the next trial. An optional break was offered after each block. Within each block, trials were presented in a random order.

### Data availability

Raw data for all experiments included in the current manuscript are available on figshare: https://figshare.com/s/58fec2b9ed9fd535f727.

## Results

For each participant and condition, we measured mean accuracy and reaction time (ms) for each condition. We then computed inverse efficiency by dividing response time by accuracy^35^. Given that participants were instructed to respond as quickly and accurately as possible, subcortical facilitation can be instantiated in reaction times, accuracy, or both. In this context, inverse efficiency is likely to provide the most sensitive measure of performance because, unlike accuracy or reaction time alone, it captures speed-accuracy trade-offs in individual participants. For this reason, we based our primary analyses and conclusions on inverse efficiency. However, for completeness, we have included additional analyses for accuracy and reaction time for each experiment (see Figure S1 and Supplementary Information). In these analyses, most effects observed for inverse efficiency were also present for reaction times, and some were observed for accuracy.

**Figure 3:**
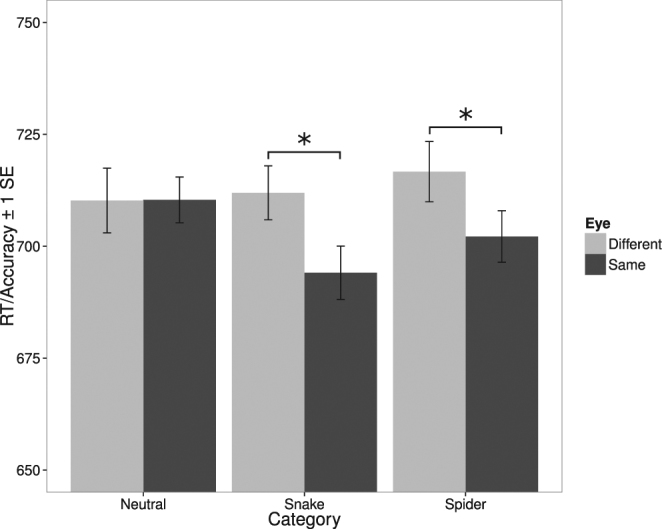
Results for Experiment 1. RT/Accuracy (inverse efficiency) as a function of image category (neutral, snake, spider), and eye condition (same, different). Error bars are within-subjects standard errors^56^. *Indicates a significant difference.

Inverse efficiency for each condition is shown in Fig. 3. We carried out a repeated-measures factorial ANOVA with eye condition (same, different) and image category (neutral, snake, spider) as within-subjects factors and inverse efficiency as the dependent variable. There was a significant main effect of eye condition, *F*(1, 34) = 8.94, *p* < 0.006, no main effect of image category, *p* > 0.70, and, importantly, a significant interaction between eye condition and image category, *F*(2, 68) = 4.08, *p* < 0.025. We followed up the significant interaction with Bonferroni-corrected (alpha = 0.017) paired-samples t-tests comparing the same and different eye conditions for each image category. The difference between eye conditions was significant for spiders, *t*(34) = 3.00, *p* < 0.005, and snakes, *t*(34) = 2.74, *p* < 0.01, but not for neutral images, *p* > 0.95. As shown in Fig. 3, for snakes and spiders, inverse efficiency was higher (indicating poorer performance) for the different eye condition than for the same condition, a result indicating that there was a monocular advantage for these two categories.

## Discussion

We observed a monocular advantage for discrimination among snakes and among spiders, but not for discrimination among neutral images. These results suggest that the monocularly segregated subcortical visual pathway selectively facilitates behavioral responses to visual threat.

We did not observe faster and/or more accurate responses for threatening images than for neutral images, as observed in previous studies^1–7^. However, the current paradigm differs from that used in the previous studies in that participants in the previous studies detected threatening or non-threatening targets in the context of non-threatening distractors, whereas participants in the current study made within-category judgments about pairs of threatening images or pairs of non-threatening images. The comparison between categories might be critical for measuring the threat superiority effect, and so the current paradigm may not be sensitive to this effect. Also, the images used in the current study were selected for emotional relevance, not for visual similarity. Hence, differences between image categories in within-category visual similarity might give rise to differences in performance between image categories, or might mask differences between categories (e.g., the threat superiority effect). Given that all hypotheses in the current study are related to the effect of a particular manipulation (e.g., monocular versus dichoptic presentation) on performance within a given image category, general differences in performance between images categories do not impact our conclusions related to these hypotheses.

## Experiment 2

The monocular advantage observed for snakes and spiders in Experiment 1 could potentially reflect subcortical facilitation of responses to any emotionally relevant stimuli. In Experiment 2, we investigated this possibility by measuring the monocular advantage for positive stimuli (pleasant nature scenes), which are emotionally relevant but not threatening.

Previous research indicates that, under at least some circumstances, adult humans detect threatening images faster than non-threatening images, whether the threatening images depict threatening animals (e.g., snakes, and spiders) or modern, man-made threats^8,9^. Hence, it is of interest whether the monocular advantage observed for threatening animals in Experiment 1 would generalize to a modern, man-made threat. In Experiment 2, we tested this possibility by measuring the monocular advantage for images of guns.

### Method

#### Participants

Participants were 36 adults (19 female, *M* age = 19.11 years, age range = 18–22 years). One additional participant was tested, but was excluded because accuracy or response time was greater than 2.5 standard deviations away from the group mean in at least one condition. All other information about participants was the same as in Experiment 1.

#### Stimuli

Stimuli were positive and neutral images from the Geneva Affective Picture Database (GAPED)^50^, and images of assault rifles from ImageNet^52^ (all ImageNet images are available at http://www.image-net.org). In the GAPED ratings, the positive images were significantly more positive in valence (*M* valence = 89.65, *SD* valence = 6.20) than the neutral, snake and spider images (*M* and *SD* for neutral, snake, and spider images given in Experiment 1), and were significantly lower in arousal (*M* arousal = 21.60, *SD* arousal = 10.72) than the snake and spider images, but did not significantly differ from the neutral images^50^. As in Experiment 1, images including written text and people were excluded from the stimulus set, and image counts were equalized to match that of the category with the smallest number of images (positive), leaving 51 images per category. Stimulus presentation settings were the same as in Experiment 1.

#### Apparatus and Procedure

The apparatus and procedure for Experiment 2 were the same as for Experiment 1.

## Results

**Figure 4:**
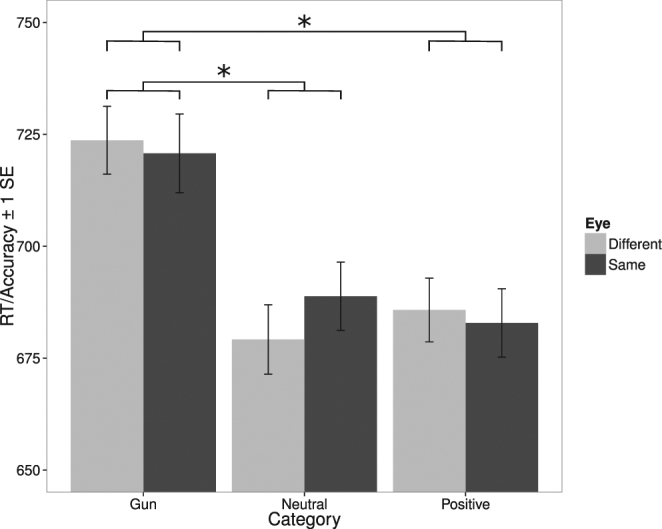
Results for Experiment 2. RT/Accuracy (inverse efficiency) as a function of image category (gun, neutral, positive), and eye condition (same, different). Error bars are within-subjects standard errors^56^.

Results for Experiment 2 are shown in Fig. 4. We carried out a repeated-measures factorial ANOVA with image category (neutral, guns, positive) and eye condition (same, different) as within-subjects factors, and inverse efficiency as the dependent variable. There was no significant main effect of eye condition, *p* > 0.75, a significant effect of image category, *F*(2, 70) = 6.50, *p* < 0.003, and no significant interaction, *p* > 0.25. We followed up the significant effect of image category with Bonferroni-corrected (alpha = 0.017) paired-samples t-tests comparing inverse efficiency between each possible pair of image categories. There were significant differences between guns and positive images, *t*(35) = 3.07, *p* < 0.005, between guns and neutral images, *t*(35) = 3.03, *p* < 0.005, but not between neutral and positive images, *p* > 0.97. As shown in Fig. 4, inverse efficiency was greater (indicating poorer performance) for guns than for positive and neutral images, with no difference between the latter two. The poorer performance observed for guns seems likely to reflect the fact that all images in that category depict highly similar objects, whereas images in the other categories depict a variety of objects, and may therefore be easier to discriminate.

To confirm that there was no monocular advantage for any of the image categories presented in the current experiment, we carried out Bonferroni-corrected (alpha = 0.017) paired-samples t-tests (one per image category) comparing inverse efficiency between the two eye conditions. There were no significant differences, *p*s > 0.15, a result confirming that there was no significant monocular advantage for any of the image categories.

To investigate whether the results for neutral images differed between the current experiment and Experiment 1, we carried out a mixed ANOVA with eye condition (same, different) as a within-subjects factor, experiment (1, 2) as a between-subjects factor, and inverse efficiency for neutral stimuli as the dependent variable. There were no significant main effects or interactions, *p*s > 0.20. This result indicates that the results for neutral images did not differ between the current experiment and Experiment 1. We also carried out corresponding analyses for accuracy and reaction time data (see Supplementary Information).

## Discussion

In the current experiment, we observed no monocular advantage for neutral images, guns, or positive images (pleasant nature scenes). The absence of a monocular advantage for positive images may suggest that the monocular advantage observed for snakes and spiders in Experiment 1 does not reflect a general process that would affect performance for any emotionally relevant stimulus, but may instead reflect a process specific to only some types of emotionally relevant stimuli. The absence of a strong monocular advantage for guns provides evidence that subcortical facilitation of responses to threat may be specific to ancestral threats.

## Experiment 3

In Experiment 3, we investigated the role of the retino-tectal subcortical pathway in facilitating behavioral responses to visual threat likely by virtue of the projections from the retina to the superior colliculus, pulvinar, and amygdala. To do so, we compared responses to images presented in the temporal and nasal hemifields. We expected that if the retino-tectal pathway selectively facilitates behavioral responses to threat, there would be a temporal hemifield advantage for snakes, with a weaker or absent temporal hemifield advantage for neutral images.

### Method

#### Participants

Participants were 37 adults (22 female, *M* age = 22.46 years, age range = 18–22 years). Two additional participants were tested, but were excluded from all analyses because they inadvertently moved the mirrors in the stereoscope during the experiment. Five additional participants were tested, but were excluded because accuracy or mean response time was greater than 2.5 standard deviations away from the group mean in at least one condition. All other information about participants was the same as in the previous experiments.

#### Apparatus

The apparatus was the same as in the previous experiments, with the exception that the mirrors were placed 2 cm closer to the participant (total viewing distance of 61.5 cm instead of 63.5 cm), which allowed us to present images further into the participant’s nasal hemifield.

#### Stimuli

Stimuli were the snake and neutral stimuli from Experiment 1. Stimuli were presented using the same parameters as in the previous experiments, with the exception that images were presented 5.26 cm (4.89°) toward the nasal and temporal visual hemifields, and were presented at a slightly smaller size (7.92 cm [7.04°] wide and 5.98 cm [5.51° at temporal side of image, 5.57° at nasal side of image] high) than in the previous experiments.

#### Design and Procedure

The design and procedure were based on those of Experiment 1 and 2, with several modifications. After completing the same calibration procedure as in Experiments 1 and 2, participants completed an additional procedure to confirm that each participant could see all stimuli that would be presented during the experiment. Participants viewed four black rectangles of the same size (2 in each eye) as the images that would be presented throughout the experiment, presented at the positions in which those images would be presented. Participants viewed the rectangles monocularly, and confirmed that they were able to see both rectangles. This procedure was completed independently for each eye.

**Figure 5:**
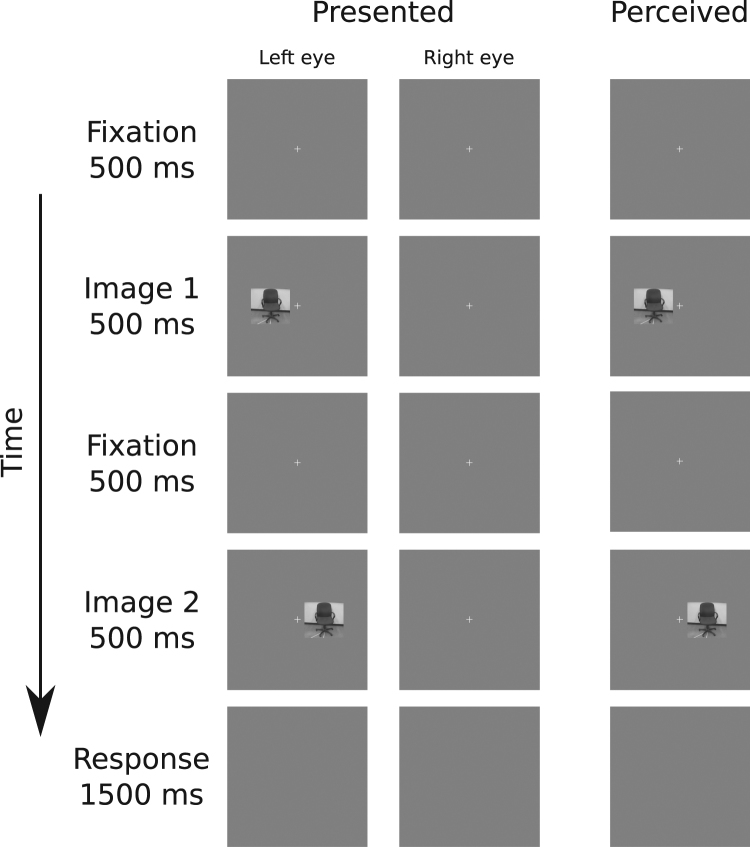
Sequence of events for an example trial in Experiment 3. In this example trial, each image was presented into a different hemifield in the left eye. The “presented” column shows the events presented to each eye, and “perceived” column shows the participant’s fused percept. The stimulus image depicted here was not part of the stimulus set presented during the experiment, and is included here for demonstration purposes only.

Participants completed two blocks for snakes and two blocks for neutral images, with the order of presentation randomized for each participant. Participants completed 80 trials per block (see Fig. 5). On each trial, participants viewed a pair of images randomly selected from all images available in the current category. A white fixation cross was presented at the beginning of each trial and remained on the screen until the blank response screen at the end of the trial. Participants were instructed to fixate a white cross throughout each trial. Unlike Experiments 1 and 2, both images in each pair were presented into the same eye, with half of the pairs presented into the left eye and the other half presented into the right eye. The visual hemifields in which the first and second images were presented varied in a two (first image: nasal, temporal) by two (second image: nasal, temporal) factorial design, with each possible combination presented on 20 trials per block. We included all possible combinations of hemifields so that the participant could not predict the location of the second image from the location of the first. However, since we were interested solely in the conditions in which the two images were presented in the same visual hemifield, we restricted our analyses to those conditions. All other details were the same as in Experiments 1 and 2.

## Results

**Figure 6:**
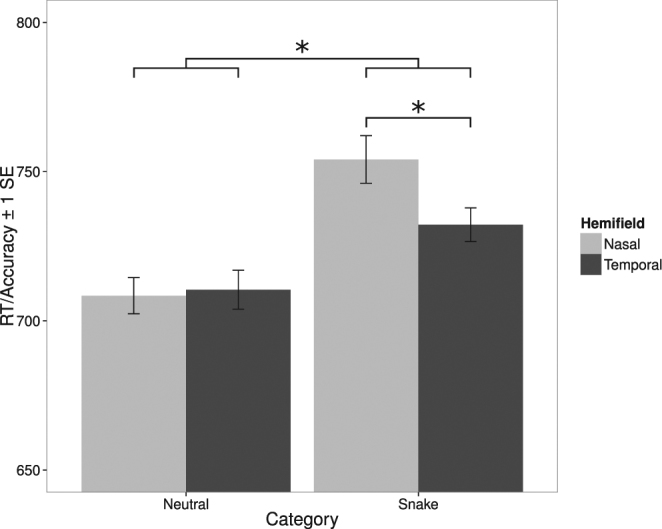
Results for Experiment 3. RT/Accuracy (inverse efficiency) as a function of image category (snake, neutral) and hemifield (nasal, temporal). Error bars are within-subjects standard errors^56^. *Indicates a significant difference.

We carried out a repeated-measures factorial ANOVA with hemifield (nasal, temporal) and image category (snake, neutral) as within-subjects variables, and with inverse efficiency as the dependent variable (see Fig. 6). There were significant main effects of image category, *F*(1, 36) = 9.62, *p* < 0.004, and a marginally significant effect of hemifield, *F*(1, 36) = 4.08, *p* = 0.051. These main effects were qualified by a significant interaction between image category and hemifield, *F*(1, 36) = 4.22, *p* < 0.05. We followed up the interaction with Bonferroni-corrected (alpha = 0.025) paired-samples t-tests comparing the two hemifields within each image category. There was a significant difference for snakes, *t*(36) = 2.54, *p* < 0.02, but not for neutral images, *p* > 0.75. As shown in Fig. 6, there was a temporal hemifield advantage in performance for snakes, but not for neutral images.

In the current experiment, but not in Experiment 1, performance, as indexed by inverse efficiency, was better for neutral images than for images of snakes. We hypothesized that the difference in performance observed in the current experiment could reflect a difference between image categories in the spatial frequency (SF) content of the images. To test this hypothesis, we ran an ideal observer analysis in which a simulated observer discriminated between pairs of images of snakes or neutral images when these images were presented in noise filtered to contain information in specific SF bands (see Supplementary Information and Figure S2). Results indicated that at low SFs (<5 cycles/degree), there was more information available to perform the task for neutral images than for the snake images, with no differences between image categories at higher SFs. In humans, sensitivity to SFs above approximately 5 cycles/degree is selectively attenuated in the near periphery^53,54^. Hence, absolute performance levels may have been better for neutral images than for snakes because the spatial frequency bands to which participants had the greatest access contained more information useful for discrimination among the neutral images than for the snake images. The difference between snakes and neutral images may not have been as prominent in Experiment 1 because images in that experiment were presented centrally, and so participants in that experiment would have had greater access to high SF information, which was equally available in the two image categories. The key finding, however, concerns the relative advantage of processing in the temporal than nasal hemifield which is only present for snakes and not for neutral images.

## Discussion

In the current experiment, performance for snakes was better in the temporal hemifield than in the nasal hemifield. For neutral images, there was no difference in performance between the two hemifields. Given that there are more projections from the nasal hemiretina (temporal visual hemifield) to the retino-tectal subcortical pathway than there are from the temporal hemiretina (nasal visual hemifield)^47,48^, these results provide evidence that the retino-tectal pathway selectively facilitates behavioral responses to visual threat.

## General Discussion

In the current study, we investigated whether the subcortical visual pathway facilitates behavioral responses to threat in adult humans, which types of threatening images receive subcortical facilitation, and which parts of the subcortical visual pathway are involved in facilitating responses to threatening stimuli. In Experiment 1, we observed a monocular advantage for snakes and spiders, but not for neutral images, a result indicating that the subcortical visual pathway facilitates behavioral responses to threatening images. In Experiment 2, we observed no monocular advantage for a modern threat (guns), positive images (pleasant nature scenes), or neutral images. This result provides evidence that the subcortical facilitation observed in Experiment 1 reflects a process that may be specific to ancestral threats. In Experiment 3, we investigated whether the retino-tectal subcortical pathway selectively facilitates processing of threat by measuring the discrimination of pairs of images presented to the nasal or temporal hemifield. We observed a temporal hemifield advantage for snakes, but not for neutral images. This result indicates that the retino-tectal subcortical visual pathway selectively facilitates behavioral responses to threat.

The monocular advantage observed for snakes and spiders in Experiment 1 and the temporal hemifield advantage for snakes in Experiment 3 provide new information about the role of the subcortical visual pathway in behavioral responses to threat in adult humans. Previous neuroimaging studies indicate that both subcortical regions are active during viewing of threatening images^16–19^, but do not provide detailed information about the relation between these neural responses and behavioral responses to threat. Our finding of a monocular advantage for snakes and spiders demonstrates that the subcortical visual pathway selectively facilitates behavioral responses to ancestral threats, and our finding of a temporal hemifield advantage for snakes suggests that this subcortical facilitation may be driven primarily by the retino-tectal subcortical visual pathway, which projects directly from the retina to the superior colliculus, pulvinar, and amygdala^13,14,40,41^. This result is in line with evidence that both pulvinar and amygdala respond selectively to ancestral threats^19,31^, and provides support for recent theoretical proposals that the superior colliculus-pulvinar pathway may be critical not only for detection of visual threat, but also for coordinating behavioral responses to threat^15^. Note, however, that the current results seem unlikely to reflect activity in amygdala alone, as the human amygdala appears to encode information about both positive and negative valence^55^, and we did not observe a monocular advantage for images with positive valence (the pleasant nature scenes presented in Experiment 2).

Our results also provide evidence consistent with theoretical proposals that processing of visual threat involves a subcortical mechanism that is at least partially conserved across phylogeny and ontogeny^11,12,14,15^. In the case of ontogeny, it has been proposed that a subcortical mechanism guides processing of emotionally relevant stimuli in infancy, but this subcortical mechanism might be supplemented or replaced by a cortical mechanism later in development^13^. Our findings for snakes and spiders suggest that this putative subcortical mechanism continues to facilitate threat processing in adulthood, and therefore suggest conservation of lower-order neural mechanisms for threat processing over ontogeny. With respect to phylogeny, it has been proposed that in both human and non-human primates, an evolved subcortical system mediates behavioral responses to threat^10–12,15^. Our findings for snakes and spiders suggest that this subcortical system is active in adult humans, and therefore provide evidence consistent with conservation of lower-order neural mechanisms for threat processing over phylogeny. However, our results also suggest at least some degree of divergence between human and non-human primates in mechanisms for processing of some specific types of ancestral threats. Whereas humans commonly fear snakes and spiders, and show enhanced behavioral performance for both types of animals^2–7,32^, non-human primates commonly fear snakes but not spiders, and show enhanced behavioral performance for snakes, but not spiders^27,28^. Hence, mechanisms underlying behavioral responses to threat may be somewhat specific to snakes in non-human primates, but appear to generalize to spiders in humans^27^. Our finding of a similar monocular advantage for snakes and spiders in Experiment 1 is consistent with the generalization to spiders in humans, and therefore provides support for at least some divergence between human and non-human primates in processing of specific types of ancestral threats.

Our results also provide new information concerning the specificity of subcortical mechanisms for processing of visual threat in adult humans. Previous work indicates that human amygdala encodes information about both positive and negative valence^50^. Hence, it is possible that the subcortical visual pathway could facilitate behavioral responses to non-threatening images with positive valence. However, the absence of a monocular advantage for positive images in Experiment 2 suggests that this is not the case. Also, our finding that the monocular advantage for snake and spiders in Experiment 1 did not generalize to guns is consistent with findings that threatening animals, but not weapons, strongly activate amygdala^19^. However, it is not consistent with findings that subjects are faster in detecting threatening images than non-threatening images (e.g., flowers), whether the threatening images depict modern (e.g., guns, syringes) or ancestral threats (e.g., snakes)^8,9^. One possible explanation for these results is that the subcortical pathway might facilitate responses to ancestral threats, whereas a cortical mechanism may facilitate responses to modern threats^19^.

Taken together, the results advance the current understanding of responses by adult humans to threat by revealing the characteristics of behaviors driven by a lower-order neural mechanism that is specialized for the processing of ancestral threats. Specifically, the findings have not only implicated subcortical structures but have elucidated the specific nature of the information mediated by the lower-order subcortical representations. The results also contribute to ongoing debates concerning the biological generality of neural mechanisms for processing of complex, emotionally-relevant stimuli^15^ by providing evidence for conservation of lower-order neural mechanisms for processing of ancestral threats across both ontogeny and phylogeny.

## Electronic supplementary material

Supplementary Information
