# Supplementary material for: Subcortical Facilitation of Behavioral Responses to Threat
Source: Sci Rep. 2017 Oct 12;7:13087. doi: 10.1038/s41598-017-13203-8 (PMC5638842; doi:10.1038/s41598-017-13203-8)
Supplement: Supplementary file 1 — Supplementary Information [file 41598_2017_13203_MOESM1_ESM.pdf]

Subcortical Facilitation of Behavioral Responses to Threat

Mark D. Vida* (markvida@andrew.cmu.edu) and Marlene Behrmann (behrmann@cmu.edu)

Department of Psychology and Center for the Neural Basis of Cognition,

Carnegie Mellon University

Supplementary Information

Reaction Time and Accuracy Data

Experiment 1. We carried out a repeated-measures factorial ANOVA with eye condition (same eye, different eye) and image category (snake, spider, neutral) as independent variables and reaction time as the dependent variable (see Figure S1 for reaction time and accuracy data for all experiments). There was a marginally significant effect of eye condition, $F(1, 34) = 4.27$, $p = .046$, no effect of image category $p > .4$, and a marginally significant interaction between image category and eye condition, $F(2, 68) = 2.88$, $p = .063$. We followed up the marginally significant interaction with Bonferroni corrected (alpha = .017) paired samples t-tests comparing the two eye conditions for each image category. There were trends toward differences between the two eye conditions for snakes, $t(34) = 2.34$, $p = .025$ and spiders, $t(34) = 2.17$, $p = .037$, but not for neutral images, $p > .65$.

We also carried out a corresponding repeated-measures ANOVA with accuracy as the dependent variable. There was a marginally significant effect of eye condition, $F(1, 34) = 3.33$, $p = .077$, a significant effect of image category, $F(2, 68) = 4.36$, $p < .02$, and no interaction, $p > .15$. We followed up the significant effect of image category with Bonferroni-corrected (alpha = .017) paired-samples tests comparing all possible pairs of image categories. Accuracy was significantly higher for neutral images than for spiders, $t(34) = 3.04$, $p < .003$, and was marginally significantly higher for neutral than for snakes, $t(34) = 2.44$, $p = .02$, with no difference between spiders and snakes, $p = .71$.

Experiment 2. We carried out a repeated-measures ANOVA with eye condition (same, different) and image category (gun, neutral, positive) as independent variables and reaction time as the dependent variable. There was a significant main effect of image category, $F(2, 70) = 5.19$, $p < .008$, with no main effect of eye condition, $F(2, 70) = 0.11$, $p > .70$, and no interaction, $F(2, 70) = 0.21$, $p > .80$. We followed-up the main effect of image category with Bonferroni-corrected (alpha = .017) paired-samples

t-tests comparing each possible pair of image categories. Reaction times were longer for guns than for positive images, $t(35) = 2.97$, $p < .006$ and neutral images, $t(35) = 2.64$, $p < .015$, with no difference between neutral and positive images, $p > .80$.

To investigate whether reaction times for neutral images differed between the current experiment and Experiment 1, we carried out a mixed ANOVA with eye condition (same, different) as a within-subjects factor, experiment (1, 2) as a between-subjects factor, and reaction time for neutral stimuli as the dependent variable. There were no significant main effects or interactions, *p*s > .25, a result indicating that the results for neutral images did not differ between Experiments 1 and 2.

We also carried out a corresponding analysis with accuracy as the dependent variable. There was a significant main effect of image category, $F(2, 70) = 5.37$, $p < .007$, with no effect of eye condition, $p > .95$ and no interaction, $p > .1$. We followed-up the main effect of image category with Bonferroni-corrected (alpha = .017) paired-samples t-tests comparing each possible pair of image categories. Accuracy was marginally significantly lower for guns than for positive images, $t(35) = 2.08$, $p = .04$, and was lower for guns than for neutral images, $t(35) = 2.73$, $p < .01$, with no difference between neutral and positive images, $p > .1$.

To investigate whether accuracy for neutral images differed between the current experiment and Experiment 1, we carried out a mixed ANOVA with eye condition (same, different) as a within-subjects factor, experiment (1, 2) as a between-subjects factor, and accuracy for neutral stimuli as the dependent variable. There was a marginally significant effect of experiment, $F(1, 69) = 3.64$, $p = .064$, with no effect of eye condition, $p > .15$, and no interaction, $p > .095$. Accuracy was slightly higher in Experiment 1 ($M = .98$, $SD = .01$) than in Experiment 2 ($M = .97$, $SD = .02$). Different subsets of neutral stimuli were presented in the two experiments, and different groups of subjects participated in the two experiments. The slightly higher accuracy in Experiment 1 could reflect a small difference in

either or both of these factors. Importantly, the absence of a significant effect of eye condition and/or an interaction between eye condition and experiment confirms that there was no significant monocular advantage for the neutral stimuli presented in Experiments 1 and 2.

Experiment 3. We carried out a repeated-measures ANOVA with hemifield (nasal, temporal) and image category (snake, neutral) as independent variables and reaction time as the dependent variable. There were significant effects of image category, $F(1, 36) = 13.20$, $p < .0009$, and hemifield, $F(1, 36) = 4.83$, $p < .04$, and a marginally significant significant interaction, $F(1, 36) = 3.85$, $p = .057$. We followed-up the marginally significant interaction with Bonferroni-corrected (alpha = .025) paired-samples t-tests comparing the two hemifields for each image category. For snakes, reaction times were significantly shorter in the temporal hemifield than in the nasal hemifield, $t(36) = 2.75$, $p < .01$. For neutral images, there was no difference between the hemifields, $p > .95$.

We also carried out a corresponding ANOVA with accuracy as the dependent variable. There were no significant main effects of image category, $p > .95$, or hemifield, $p > .50$, and there was no interaction, $p > .20$.

Ideal Observer Analysis

The results of our ideal observer analysis are shown in Figure S2. Our ideal observer was the same as that used in a previous study[1], with two exceptions: first, the ideal observer's task was adjusted to match that of participants in Experiment 3. On each trial, input to the ideal observer was two randomly selected images from one of the image categories presented in Experiment 1. The ideal observer compared a noisy version of the first image (stimulus) in the pair to noisy versions of each image in the pair (templates). Noise was randomly generated for each image and trial. The ideal observer decided that the stimulus matched whichever template was most similar to the stimulus. Second, higher spatial frequencies were included in the current analysis than in Vida and Maurer[1]. The

following spatial frequencies were tested in the current study: 3.6, 5.1, 7.1, 10.1, 14.3, 20.2 28.6, 40, 47.1, 80.8, 114.3, 161.6, 228.6 323.2, 457.1 cycles/image width (0.5, 0.7, 1.0, 1.4, 2.0, 4.1, 5.7, 8.1, 11.5, 16.2, 22.9, 32.5, 46.9, 64.9 cycles/degree).

References

1. Vida, M. D. & Maurer, D. A comparison of spatial frequency tuning for judgments of eye gaze and facial identity. *Vis. Res.,* **112,** 45-54 (2015).

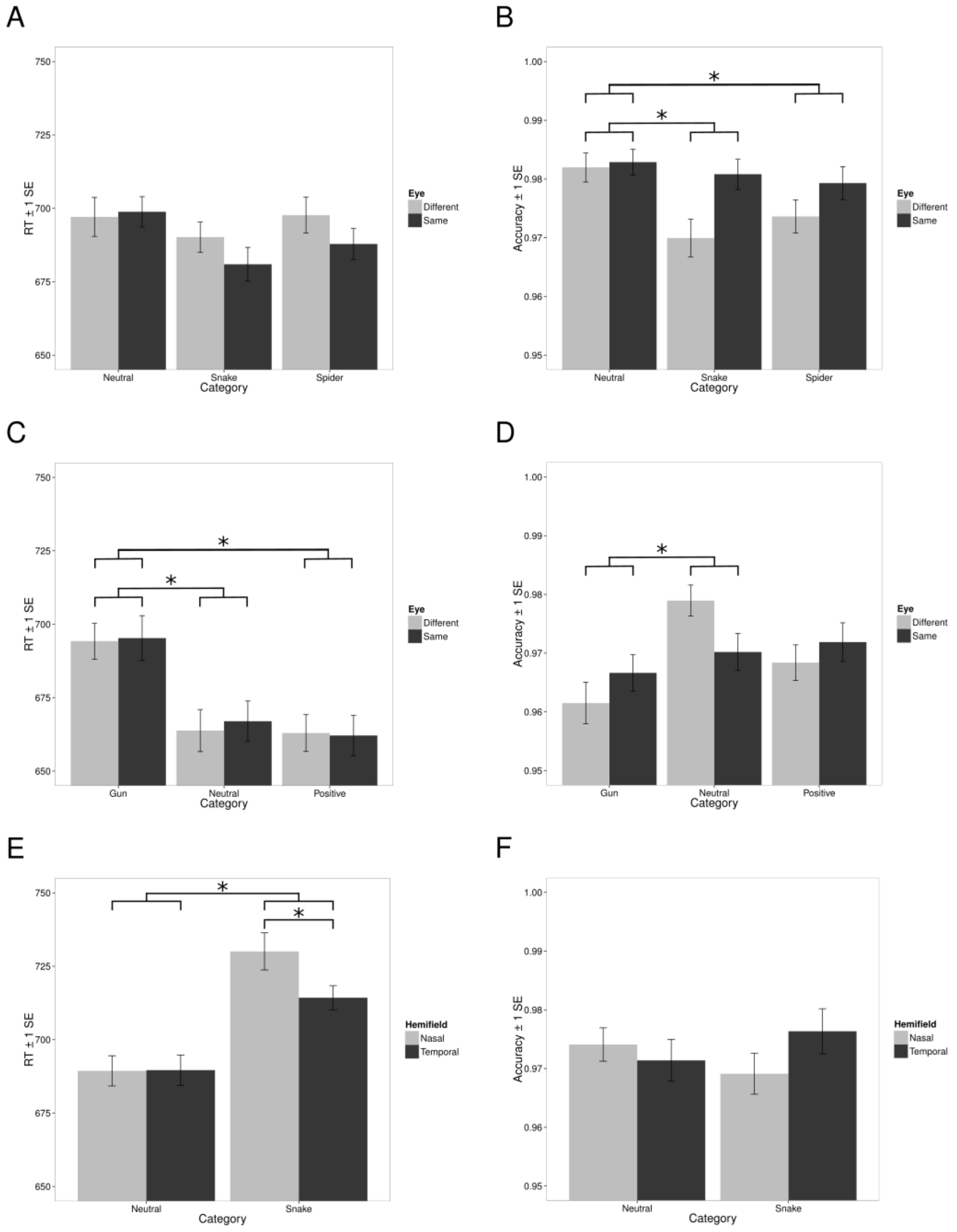

Figure S1. Reaction time and accuracy data for all experiments. Error bars are within-subjects standard errors[56]. * indicates a significant difference. A) Reaction time data for Experiment 1. B) Accuracy data for Experiment 1. C-D) Reaction time and accuracy data for Experiment 2. E-F) Reaction time and accuracy data for Experiment 3.

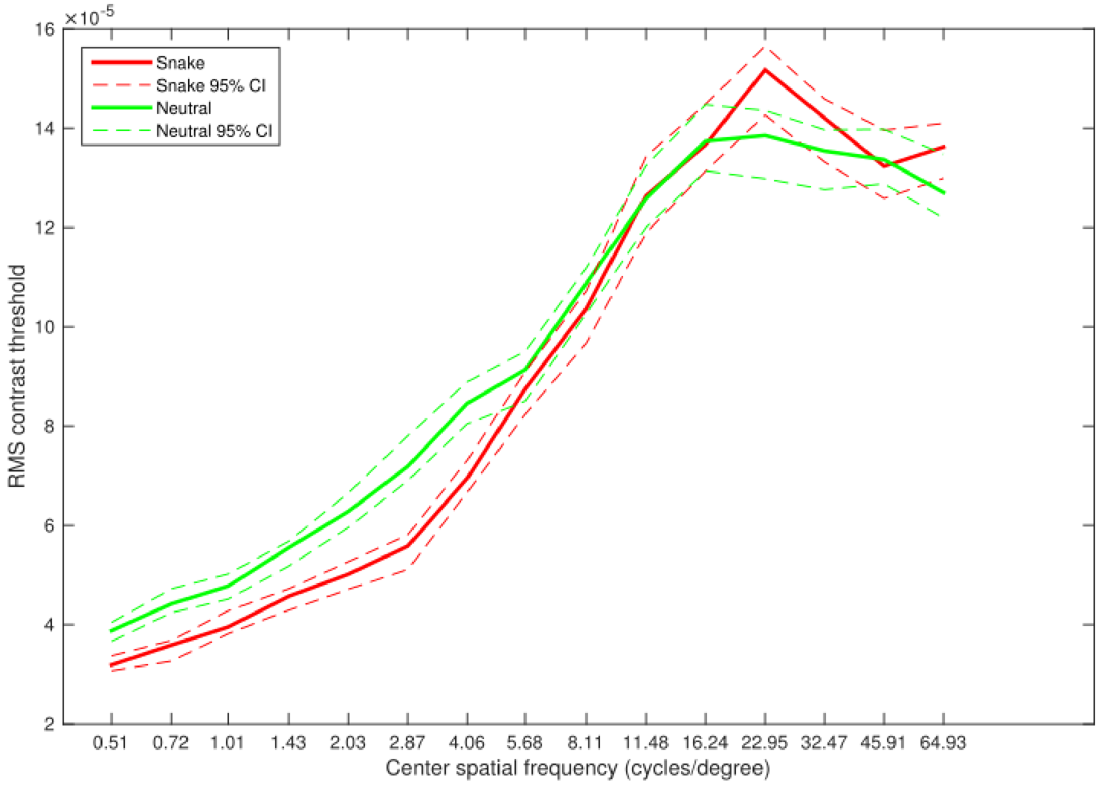

Figure S2. RMS contrast threshold for ideal observer, as a function of center spatial frequency of noise mask (cycles/degree). Higher RMS contrast threshold for a given spatial frequency band indicates greater availability of information at that spatial frequency. Dashed lines show bootstrapped 95% confidence intervals.
